# What is the association between secondhand smoke (SHS) and possible obstructive sleep apnea: a meta-analysis

**DOI:** 10.1186/s12940-022-00868-6

**Published:** 2022-06-16

**Authors:** Chen-Wei Chang, Ching-Hsiung Chang, Hung-Yi Chuang, Han-Yun Cheng, Chia-I Lin, Hsiang-Tai Chen, Chen-Cheng Yang

**Affiliations:** 1 Department of Occupational and Environmental Medicine, Kaohsiung Municipal Siaogang Hospital, Kaohsiung Medical University, Kaohsiung City, Taiwan; 2 Department of Occupational and Environmental Medicine, Kaohsiung Medical University Hospital, Kaohsiung Medical University, Kaohsiung City, Taiwan; 3 Division of Gastroenterology, Department of Internal Medicine, Kaohsiung Medical University Hospital, Kaohsiung Medical University, Kaohsiung City, Taiwan; 4 Department of Public Health and Environmental Medicine, and Research Center for Environmental Medicine, Kaohsiung Medical University, Kaohsiung City, Taiwan; 5 Health Management Center, Kaohsiung Municipal Ta-Tung Hospital, Kaohsiung Medical University, Kaohsiung City, Taiwan; 6 College of Health and Medicine, University of Tasmania, Hobart, Australia; 7 Research Center for Environmental Medicine, Kaohsiung Medical University, Kaohsiung City, Taiwan

**Keywords:** Secondhand smoke, Obstructive sleep apnea, Meta-analysis, Occupational environmental medicine, Family medicine

## Abstract

**Background:**

Association between smoking and sleep apnea is well-known from previous studies. However, the influence of secondhand smoke (SHS), which is a potential risk factor of obstructive sleep apnea (OSA), remains unclear. Our aim was to investigate the relationship between SHS and OSA using a meta-analysis.

**Materials and methods:**

For the meta-analysis, searches were performed in MEDLINE, EMBASE, and Web of Science databases on January 10, 2022, by combining various keywords including “SHS exposure” and “OSA”. Data were extracted using defined inclusion and exclusion criteria. Fixed-effects model meta-analyses were used to pool risk ratio (RR) estimates with their 95% confidence intervals (CI). I^2^ was used to assess heterogeneity. Moreover, we performed subgroup meta-analyses of children-adults, and smoker fathers and mothers.

**Results:**

In total, 267 articles were obtained through an electronic search. Twenty-six articles were included in our analysis according to the inclusion and exclusion criteria. We found evidence of an association between SHS exposure and possible OSA (RR 1.64, 95% CI 1.44–1.88). The results of the subgroup analyses showed that children passive smokers (RR 1.84, 95% CI 1.60–2.13) were at greater risks of possible OSA than adult passive smokers (RR 1.35, 95% CI 1.21–1.50). Also, significant differences were observed in mothers with smoking exposure (RR 2.61, 95% CI 1.62–4.21, *p* < 0.0001), as well as in fathers with smoking exposure (RR 2.15, 95% CI 0.98–4.72, *p* = 0.06).

Short conclusion.

Our meta-analysis confirmed that SHS exposure is significantly associated with OSA. In the subgroup analyses, the association of SHS and possible OSA was significant in both children and adults, as well as in smoker mothers and fathers.

## Highlights

1. This is a meta-analysis to evaluate the relationship between secondhand smoke (SHS) exposure and obstructive sleep apnea (OSA) in both adults and children.

2. Our meta-analysis revealed a significantly positive association between SHS exposure and possible OSA in children and adults.

3. Both smoking in mothers and fathers are associated with significantly higher risk of OSA in children.

## Background

Sleep apnea syndrome (SAS) is pathological breathing characterized by repetitive airflow cessation or reduction, causing intermittent hypoxemia or arousal during sleep. There are two major forms of SAS, the central sleep apnea (CSA) and obstructive sleep apnea (OSA); of these, OSA is the most common [1]. The prevalence rate of OSA in men and women is 27.3% and 22.5%, respectively [2]. OSA has multiple effects on various organ systems, including metabolic, neuropsychiatric, as well as cardiovascular systems. In the cardiovascular system, OSA shows effects on the occurrence of hypertension, coronary artery disease, stroke, heart failure, as well as arrhythmias [3]. The well-known pathogenesis of OSA is an abnormality of the upper airway and dysfunction of the local dilator muscles. Furthermore, some risk factors contributing to OSA include age, male sex, and obesity [4]. The most important is that OSA significantly increases all-cause mortality as well as cardiovascular mortality has been declared in several meta-analysis studies [5, 6].

Tobacco use is one of the important health hazards worldwide, contributing to more than 7 million deaths per year [7]. Among tobacco exposure, cigarette smoking is one of the most common. Thousands of chemicals and carcinogens enter the human body by inhaling cigarette smoke, causing several comorbidities, such as airway disorders, metabolic diseases, cardiovascular diseases, and various types of cancers [8].

Secondhand smoke (SHS), alternatively referred to as passive smoking, indicates the inhalation of particles produced from the combustion of tobacco smoked by another person. Compared with the mainstream tobacco smoke, higher concentrations of toxic components are reported in the undiluted side stream [9]. In non-smokers, diseases caused by SHS is a major concern, including in the pediatric population, and these are seldom active smokers [10, 11].

In an observational study, a strong association was reported between smoking and sleep apnea [12]. Włodarska et al. pointed out children exposed to SHS have higher risk of OSA [13], while Sogut et al. showed no significant association between SHS and OSA [14]. However, the association between SHS and OSA remains inconsistent, and the related literature is scarce. Therefore, our purpose was to determine the association between SHS and OSA using a meta-analysis.

## Material and method

### Protocol and registration

According to the Preferred Reporting Items for Systematic Reviews and Meta-Analyses (PRISMA) guidelines, we carried out a systematic review and meta-analysis. This review protocol is registered at PROSPERO (registration number, CRD42020191098) and Kaohsiung Medical University Hospital Institutional Review Broad (KMUHIRB-EXEMPT(II)-20,220,004).

### Data sources and search terms

MEDLINE (PubMed), Embase, and Web of Science databases were queried on January 10, 2022, for related studies. There were no limitations on the publication dates, besides, the target key words used to identify all the articles. Two researchers (C–C Yang and H-Y Cheng) performed a rudimentary search using different key words. The researchers separately proposed a set of key search words as follows: "Pollution, Tobacco Smoke" OR "Pollutions, Tobacco Smoke" OR "Smoke Pollution, Tobacco" OR "Smoke Pollutions, Tobacco" OR "Tobacco Smoke Pollutions" OR "Environmental Tobacco Smoke Pollution" OR "Environmental Smoke Pollution, Tobacco" OR "Air Pollution, Tobacco Smoke" OR "Environmental Pollution, Tobacco Smoke" OR "Smoking, Passive" OR "Passive Smokings" OR "Smokings, Passive" OR "Secondhand Smoking" OR "Secondhand Smokings" OR "Smoking, Secondhand" OR "Smokings, Secondhand" OR "Second Hand Smoke" OR "Hand Smoke, Second" OR "Hand Smokes, Second" OR "Second Hand Smokes" OR "Smoke, Second Hand" OR "Smokes, Second Hand" OR "Secondhand Smoke" OR "Secondhand Smokes" OR "Smoke, Secondhand" OR "Smokes, Secondhand" OR "Involuntary Smoking" OR "Involuntary Smokings" OR "Smoking, Involuntary" OR "Smokings, Involuntary" OR "Passive Smoking" OR "Tobacco Smoke Pollution"[Mesh] AND “Apnea Syndrome, Sleep” OR “Apnea Syndromes, Sleep” OR “Sleep Apnea Syndrome” OR “Sleep Hypopnea” OR “Hypopnea, Sleep” OR “Hypopneas, Sleep” OR “Sleep Hypopneas” OR “Apnea, Sleep” OR “Apneas, Sleep” OR “Sleep Apnea” OR “Sleep Apneas” OR “Sleep Apnea, Mixed Central and Obstructive” OR “Mixed Central and Obstructive Sleep Apnea” OR “Sleep Apnea, Mixed” OR “Mixed Sleep Apnea” OR “Mixed Sleep Apneas” OR “Sleep Apneas, Mixed” OR “Hypersomnia with Periodic Respiration” OR “Sleep-Disordered Breathing” OR “Breathing, Sleep-Disordered” OR “Sleep Disordered Breathing” OR “Apneas, Obstructive Sleep” OR “Obstructive Sleep Apneas” OR “Sleep Apneas, Obstructive” OR “Obstructive Sleep Apnea Syndrome” OR “Obstructive Sleep Apnea” OR “OSAHS” OR “Syndrome, Sleep Apnea, Obstructive” OR “Sleep Apnea Syndrome, Obstructive” OR “Apnea, Obstructive Sleep” OR “Sleep Apnea Hypopnea Syndrome” OR “Syndrome, Obstructive Sleep Apnea” OR “Upper Airway Resistance Sleep Apnea Syndrome” OR “Syndrome, Upper Airway Resistance, Sleep Apnea” OR “Snoring” OR "Sleep Apnea, Obstructive"[Mesh] OR "Sleep Apnea Syndromes"[Mesh]. The appropriate modified search methods were performed for EMBASE and Web of Science databases.

### Eligibility criteria

Inclusion criteria of the study are: (1) exposure was SHS; and (2) based on questionnaire assessment or polysomnography (PSG); the outcome was a possible OSA.

### Study selection process

In the first screening, two investigators (H-Y Cheng, and C–C Yang) individually assessed the abstracts of the preliminary articles included. Then in the second screening, two investigators (C-W Chang, and C-H Chang) performed the full text screening to identify articles that met the eligibility criteria and excluded those that were not eligible. Disagreements between C-W Chang and C-H Chang about the eligibility of a study were resolved by three researchers (H-Y Chuang, C-I Lin, and H-T Chen) following a comprehensive evaluation.

### Data collection

From each eligible study, we extracted information regarding the study characteristics, SHS, possible OSA cases, and the association between SHS and possible OSA. If this information was missing or inaccurate, we tried to reach the relevant authors to provide clearer details.

### Study characteristics

We recorded the following data in respect of the study characteristics: the country where the study was done, publication year, sampling framework (clinical- or community-based), sample size, characteristics of participants, number of the outcome events (i.e., the number of possible OSA), as appropriate.

### Secondhand smoke (SHS)

We defined SHS as “passive smoking” and “involuntary smoking”, including the composition of various complex mixtures from the smoldering end of tobacco, known as side stream smoke (SSS), and from the smoker's exhaled smoke and minor amounts of smoke that escape during the puff-drawing [9].

### Possible sleep apnea syndrome cases

The classification of possible OSA are as below: questionnaires for OSA risk assessment, snoring, or PSG evaluation. Possible OSA was defined based on individual studies.

### Statistical analysis

We calculated the overall pooled prevalence risk ratios (RRs) from possible SAS cases according to SHS and non-SHS exposures. Using the 95% confidence interval (CI) for the RR, we appraised the standard error (SE) for the RR. In this meta-analysis, we reported the prevalence RR and SE. The main prevalence RRs and the SEs were combined using a fixed-effects model meta-analysis, to calculate the pooled prevalence RR and its 95% CI for the primary outcome. We built a fixed-effects model to assess the possibility of heterogeneity regarding whether the RRs of the included studies originated from their characteristics [15]. I^2^ was applied for reporting the heterogeneity among these enrolled studies. Moreover, separate subgroup meta-analysis for children-adults, and smoker fathers and mothers, were performed. Review Manager version 5.4 and R version 3.6.2 were used for all statistical analysis.

## Results

### Selected studies

**Fig. 1:**
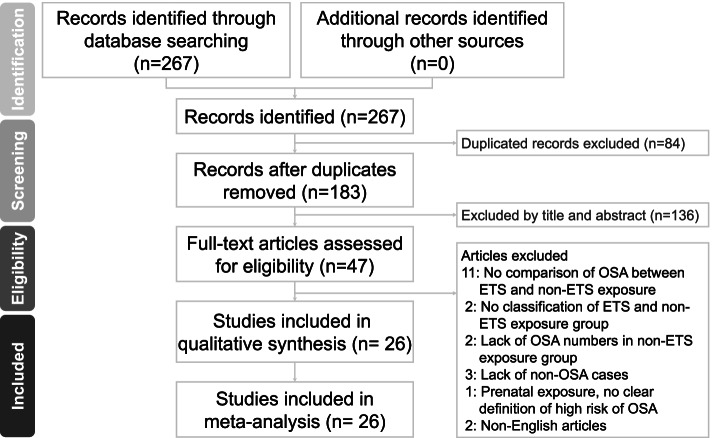
Preferred Reporting Items for Systematic Reviews and Meta-Analyses flow diagram

The summary of the present literature search procedure is shown in Fig. 1. The data base search was from three different databases (PubMed, EMBASE and Web of Science), which gave a result of 267 articles. However, 84 article were removed due to duplication, therefore a total of 183 studies were screened for abstracts and title. At the first phase of the screening processes, 136 studies were excluded leaving 47 studies for full text screening. In the second phase of the screening 21 studies did not meet the inclusion criteria due to inappropriate study design, insufficient patient group, inappropriate patient group and non-English studies. Finally, we included 26 studies with 115,080 participants, in both the narrative synthesis and meta-analysis.

### Study characteristics

Table 1 presents the 26 studies that met our inclusion criteria [13, 14, 16–39], and 23 used cross-sectional study design [13, 14, 16–24, 26, 28–30, 32–39]. The remaining three studies, categorized into exposure vs. non-exposure groups, used retrospective cohort (Huang et al.) [25]; prospective cohort (Kannan et al.) [27]; and case–control (Nosetti et al.) [31] study designs. The assessment of OSA in 21 studies [16, 18–24, 26–30, 32–39] was questionnaire-based, while in five studies [13, 14, 17, 25, 31], assessment was via PSG. The study participants in 23 studies [13, 14, 16–21, 23, 24, 26–31, 33–39] were children; three of the remaining studies were conducted in adults [22, 25, 32].

**Table 1:** Studies included in the systematic review and meta-analysis (*N* = 26)

First author (year), country	Study design	N	Recruitment	Participants	Sex	Exposure variable	Outcome measures	Number of outcome events/ cases	Comparison
Anuntaseree (2001), Thailand	Cross-sectional	1142	Community	Children aged 6–13 years	Boys and girls	Household smoking	Ali’s questionnaire	Smoking in household: 47; non-smoking in household: 38	Habitual snoring: most nights
Brunetti (2001), Italy	Cross-sectional	1207	Community	Children aged 3–11 years	Boys and girls	Passive smoking	PSG	Passive smoking: 4; non passive smoking: 10	AHI > 3
Castronovo (2002), Australia	Cross-sectional	604	Community	Children aged 2–8 years	Boys and girls	Passive smoking	Snoring questionnaire	Passive smoking: 80; non passive smoking: 74	Habitual snoring: always and often
Corbo (1989), Italy	Cross-sectional	940	Community	Children aged 6–13 years	Boys and girls	Parental smoking	Snoring questionnaire	Parental smoking exposure: 82; non parental smoking exposure: 36	Habitual snoring: snoring often
Corbo (2001), Italy	Cross-sectional	2209	Community	Children aged 10–15 years	Boys and girls	Parental smoking	Snoring questionnaire	Parental smoking exposure: 89; non parental smoking exposure: 34	Habitual snoring: snoring often
Ersu (2004), Turkey	Cross-sectional	2147	Community	Children aged 5–13 years	Boys and girls	Paternal smoking	Brouillette’s questionnaire	Paternal smoking exposure: 21; non- paternal smoking exposure: 5	High risk of OSAS: the questionnaire criteria by Brouillette et al
Franklin (2004), Sweden	Cross-sectional	15,555	Community	Adult (25–54 years)	Men and women combined	Passive smoking	Basic Nordic Sleep Questionnaire	Passive smoking exposure: 86; non-passive smoking exposure: 807	Habitual snoring: loud and disturbing snoring at least 3 nights a week
Gill (2012), New Zealand	Cross-sectional	823	Community	Children aged 3 years 0 months to 3 years 12 months	Boys and girls	Household smoking	Goldstein’s questionnaire	NR	Habitual snoring: often (4–6 night/week) or always (every night/day)
Gozal (2008), USA	Cross-sectional	16,321	Community	Children aged 5–7 years	Boys and girls	Household smoking	Gozal’s sleep questionnaire	Household smoking: 666; non household smoking: 1178	Habitual snoring: almost always (> 4 nights/week) or always on snoring frequency and medium loud to loud on loudness of snoring
Huang (2019), China	Retrospective	209	Clinic	Older than 18 years	Men and women combined	SHS	PSG	SHS exposure: 87; non- SHS exposure: 61	AHI≧5
Kaditis (2004), Greece	Cross-sectional	3666	Community	Children and adolescents aged 1–18 years	Boys and girls	Passive smoking	Brouilette’s questionnaire	Passive smoking exposure:106; non passive smoking exposure: 48	Habitual snoring: snoring every night
Kannan (2017), USA	Prospective cohort	609	Community	Child at age 4	Boys and girls	SHS	Snoring questionnaire	SHS exposure: 52; non-SHS exposure: 77	Habitual snoring ≧ 3 nights/week
Kheirandish-Gozal (2014), Iran	Cross-sectional	6000	Community	Children aged 6–12 years	Boys and girls	Parental smoking	Gozal’s sleep questionnaire	NR	Habitual snoring ≧ 3 nights/week
First author (year), country	Study design	N	Recruitment	Participants	Sex	Exposure variable	Outcome Measures	Number of outcome events/ cases	Comparison
Kuehni (2008), UK	Cross-sectional	3245	Community	Children aged 1–4 years	Boys and girls	Parental smoking	Corbo’s questionnaire	Parental smoking exposure: 241; non-parental smoking exposure: 290	Habitual snoring: snoring almost always
Li AM (2010), Hong Kong	Cross-sectional	9172	Community	Children aged 5–14 years	Boys and girls	Household smoking	Hong Kong Children Sleep Questionnaire	Household smoking: 143; non household snoring: 311	Habitual snoring: frequently, three nights or more per week
Li S (2010), China	Cross-sectional	20,152	Community	Children aged 5.08–11.99 years	Boys and girls	Household passive smoking	Children’s Sleep Habits Questionnaire	Household passive smoking: 666; non household passive smoking: 1750	Habitual snoring: frequently (2–4 nights/week) or always (5–7 nights/week)
Nosetti (2011), Italy	Case–control	1610	Community	Children aged 0–14 years	Boys and girls	SHS	PSG	SHS exposure: 274; non-SHS exposure: 306	AHI (NR)
Ohida (2007), Japan	Cross-sectional	19,386	Clinic	Pregnant women	Women	SHS	7 items questionnaire	SHSexposure: 10,269; non-SHS exposure: 9023	Snoring (often or always)
Owen (1996),	Cross-sectional	245	Community	Children aged 0–11 years	Boys and girls	Parental smoking	Owen’s questionnaire	Parental smoking exposure: 39; non parental smoking exposure: 27	Snoring (sometimes or often)
Sahin (2009), Turkey	Cross-sectional	1605	Community	Children aged 7–13 years	Boys and girls	Household smoking	Ersu’s questionnaire	Household smoking: 28; non household smoking: 14	Habitual snoring: frequently or almost every day
Sogut (2005), Turkey	Cross-sectional	1344	Community	Children aged 3–11 years	Boys and girls	Passive smoking	PSG	Passive smoking: 11; non passive smoking: 4	AHI > 3
Sogut (2009), Turkey	Cross-sectional	1030	Community	Adolescents aged 12–17	Boys and girls	Exposure to father smoking	55-item questionnaire	Exposure to father smoking: 25; non-father smoking: 16	Habitual snoring: often and always
Urschitz (2004), Germany	Cross-sectional	1760	Community	Children aged 9 ± 0.7 years	Boys and girls	Household smoking	Sleep-disordered breathing questionnaire	Household smoking: 62; non household smoking: 50	Habitual snoring: frequently and always
Włodarska (2020), Poland	Cross-sectional	160	Community	Children aged 6–18 years	Boys and girls	Exposure to tobacco smoke at home	PSG	Exposure group: 17; non exposure group: 3	Children aged < 13 years, AHI > 1.5; children aged > 13, AHI > 5
Zhang (2004), Australia	Cross-sectional	985	School	Children aged 4–12 years	Boys and girls	Household smoking	Rumchev’s questionnaire	Household smoking exposure: 84; non-household smoking exposure: 65	Habitual snoring more than 4 times/week
Zhu (2013), Hong Kong	Cross-sectional	2954	Community population	Preschool children aged 2–6 years	Boys and girls	Household smoking	The Hong Kong Paediatric Sleep Survey	household smoking exposure: 52; non-household smoking exposure: 68	Habitual snoring ≧ 3 nights/week

*SHS* Secondhand Smoke, *AHI* Apnea–Hypopnea Index, *PSG* Polysomnography, *NR* Not reported

### Results of individual studies

Table 2 shows the reported measures for the association between SHS exposure and possible OSA. Sixteen studies [13, 19–22, 24–28, 30–32, 36, 38, 39] reported a significant association between SHS exposure and possible OSA. However, the remaining 10 studies, including Sogut et al. [14] and Zhu et al. [37], revealed a negative association of SHS exposure with possible OSA.

**Table 2:** Measures of the association between secondhand smoke and high risk obstructive sleep apnea in the 26 included studies

First author (year/ journal), country	Sex	Comparison	Pooled RR	95% CI (low)	95% CI (high)	Source
Anuntaseree (2001), Thailand	Boys and girls	Habitual snoring vs. never snoring	1.35	0.95	1.91	Table 2 calculation
Brunetti (2011), Italy	Boys and girls	AHI > 3	1.65	0.53	5.18	Table 3 calculation
Castronovo (2002), Australia	Boys and girls	Habitual snoring vs. never snoring	1.09	0.84	1.41	Table 1 calculation
Corbo (1989), Italy	Boys and girls	Habitual snoring vs. non snoring	1.72	1.51	1.96	Table IV calculation
Corbo (2001), Italy	Boys and girls	Habitual snoring vs. non snoring	1.55	1.06	2.27	Table 2 calculation
Ersu (2004), Turkey	Boys and girls	High risk OSA: Brouillette’s questionnaire OSA scores > 3.5	1.55	1.13	2.13	Table 3 calculation
Franklin (2004), Sweden	Men and women combined	Habitual snoring: loud and disturbing snoring at least 3 nights a week	1.49	1.30	1.70	Table 1 and Results 2^nd^ paragraph calculation
Gill (2012), New Zealand	Boys and girls	Habitual snoring: often (4–6 night/week) or always (every night/day)	1.60	0.89	2.87	Table 3 calculation
Gozal (2008), USA	Boys and girls	Habitual snoring: almost always (> 4 nights/week) or always on snoring frequency and medium loud to loud on loudness of snoring	1.34	1.22	1.47	Table 1 calculation
Huang (2019), China	Men and women combined	AHI≧5	1.35	1.13	1.62	Table 3 calculation
Kaditis (2004), Greece	Boys and girls	Habitual snoring: snoring every night	1.89	1.35	2.64	Table 5 calculation
Kannan (2017), USA	Boys and girls	Habitual snoring ≧ 3 nights/week	2.00	1.48	2.70	Table 2 calculation
Kheirandish-Gozal (2014), Iran	Boys and girls	Habitual snoring ≧ 3 nights/week	2.25	1.41	3.59	Table 4 calculation
Kuehni (2008), UK	Boys and girls	Habitual snoring: snoring almost always	1.67	1.43	1.95	Table 1 calculation
Li AM (2010), Hong Kong	Boys and girls	Habitual snoring: frequently, three nights or more per week	0.93	0.77	1.12	Table 3 calculation
Li S (2010), China	Boys and girls	Habitual snoring: frequently (2–4 nights/week) or always (5–7 nights/week)	1.16	1.06	1.26	Table 1 calculation
Nosetti (2011), Italy	Boys and girls	AHI (NR)	2.31	2.04	2.61	Abstract calculation
Ohida (2007), Japan	Women	Snoring (often or always)	1.26	1.05	1.51	Table 2 calculation
Owen (1996), UK	Boys and girls	Snoring (sometimes or often)	1.83	1.20	2.79	Table 3 calculation
Sahin (2009), Turkey	Boys and girls	Habitual snoring: frequently or almost every day	1.79	0.96	3.34	Table 1 calculation
Sogut (2005), Turkey	Boys and girls	AHI > 3	2.19	0.70	6.85	Table 4 calculation
Sogut (2009), Turkey	Boys and girls	Habitual snoring: often and always	1.56	0.85	2.87	Table 3 calculation
Urschitz (2004), Germany	Boys and girls	Habitual snoring: frequently and always	1.29	0.91	1.83	Table 2 calculation
Włodarska (2020), Poland	Boys and girls	OSA: children aged < 13 years, AHI > 1.5; children aged > 13, AHI > 5	4.41	1.34	14.48	Table 1 calculation
Zhang (2004), Australia	Boys and girls	Habitual snoring more than 4 times/week	1.50	1.13	2.00	Table 2 and Table 3 calculation
Zhu (2013), Hong Kong	Boys and girls	Habitual snoring ≧ 3 nights/week	1.34	0.95	1.89	Table 1 calculation

*RR* Risk ratio, *CI* Confidence interval, *AHI* Apnea–Hypopnea Index, *PSG* Polysomnography, *NR* Not reported

Three studies [14, 21, 39] performed additional or sub-group analysis. For instance, Ersu et al. [21] classified SHS exposure into maternal and paternal smoking groups and compared the high-risk snorer versus never-snorer based on environmental smoking exposure of different family members. Participants with high-risk snoring are significantly associated with exposures to both maternal and paternal smoking (odds ratio [OR] 3.3, 95% CI, 1.5–7.5 versus OR 3.4, 95% CI, 1.3–9.2, respectively). Kuehni et al. [39] classified exposure to parental smoking as one and both parents smoking. Compared with one parent smoking versus none, both parents smoking versus none had significantly higher risk of habitual snoring in the fully adjusted model (OR 2.06, 95% CI, 1.48–2.87, *p* value < 0.001). Sogut et al. [14] classified smoking exposure based on the fathers’ and mothers’ smoking status. Exposure to mothers’ smoking exposure was found to be significantly more in habitual snorers than in never snored group (OR 2.3, 95% CI 1.1–4.4, *p* value < 0.05).

### Meta-analysis

**Fig. 2:**
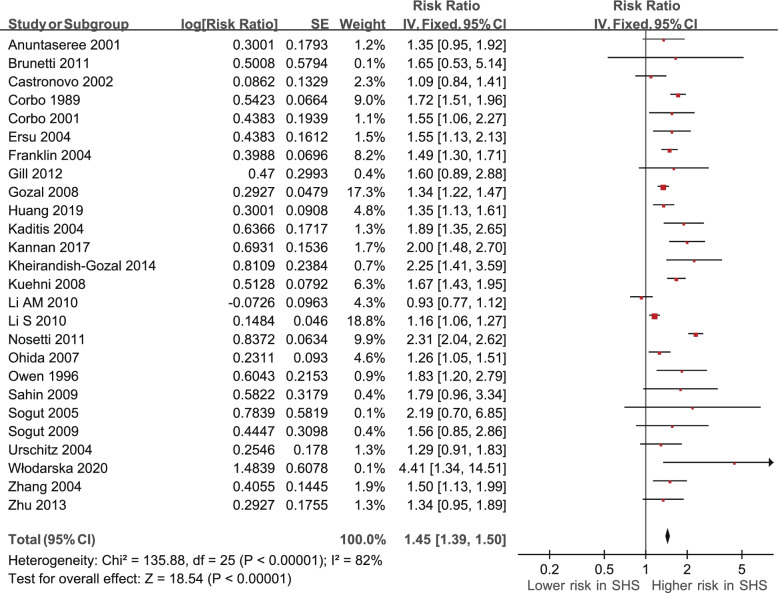
Secondhand smoke (SHS)and relative risks of obstructive sleep apnea (OSA) in the 26 studies: a fixed-effect model. CI, confidence interval

A fixed-effect model meta-analysis revealed variations in the association between exposure to SHS and possible OSA (RRs derived from 26 studies) (Table 2, Fig. 2). The pooled prevalence was significant. A fixed model meta-analysis indicated significantly positive association between exposure to SHS and possible OSA (RR 1.45; 95% CI 1.39 to 1.50, *p* < 0.00001).

**Fig. 3:**
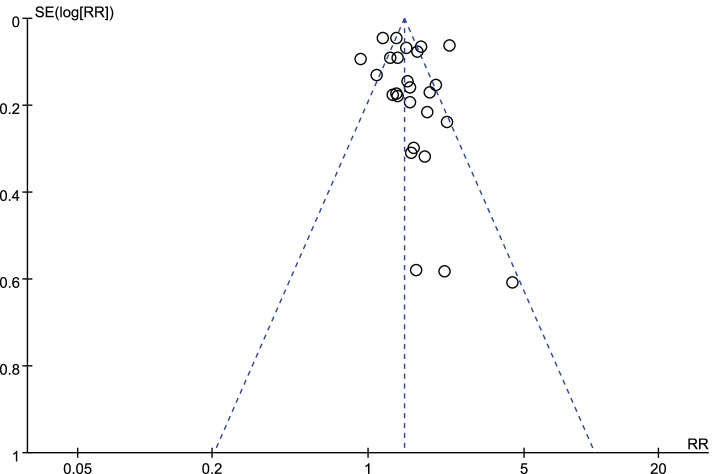
Funnel plot of the log-transformed relative risks (RRs) of obstructive sleep apnea (OSA) associated with secondhand smoke (SHS) and standard errors for the 26 studies

A funnel plot of the log-transformed RRs of the association of possible OSA with exposure to SHS as well as the SEs of the 26 RRs showed adequate number of studies had small SEs (i.e., larger sample sizes) and smaller RRs (Fig. 3).

### Subgroup analysis

**Fig. 4:**
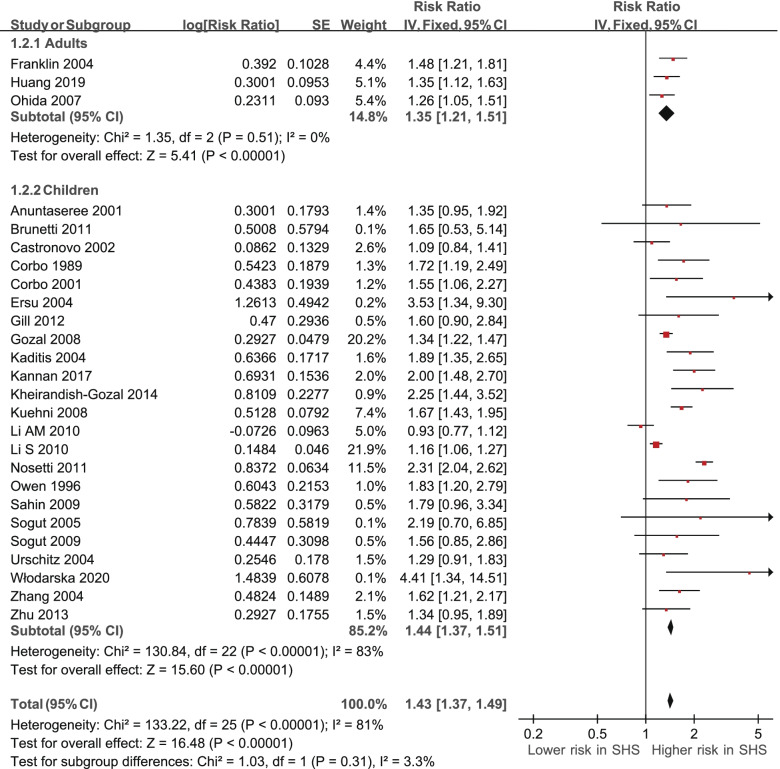
Subgroup analysis of the risk ratio of obstructive sleep apnea (OSA) by adults and children based on secondhand smoke (SHS) exposure. CI, confidence interval

We performed subgroup analyses for adults and children using fixed-effects model meta-analysis of the pooled prevalence RRs (Table 3, Fig. 4). For the adults (three RRs were derived from three studies), the pooled prevalence RR of 1.35 (95% CI 1.21 to 1.51, *p* < 0.00001) [22, 25, 32] was significant. There was low, non-significant heterogeneity (I^2^ = 0%, χ^2^ = 1.35, *p* = 0.51). For the children (23 RRs were derived from 23 studies), the pooled prevalence RR of 1.44 (95% CI 1.37 to 1.51, *p* < 0.00001) was significant. There was substantial, significant heterogeneity (I^2^ = 83%, χ^2^ = 130.84, *p* < 0.00001) [13, 14, 16–21, 23, 24, 26–31, 33–39].

**Table 3:** Subgroup analysis of the risk ratio for adults or children exposed to secondhand smoke (SHS)

Subgroup	Pooled risk ratio	95% confidence interval
**Study participants**		
**Adults**		
Franklin (2004), Sweden	1.48	1.21 - 1.81
Huang (2019), China	1.35	1.12 - 1.61
Ohida (2007), Japan	1.26	1.05 - 1.51
**Subtotal**	**1.35**	**1.21** - **1.50**
**Children**		
Anuntaseree (2001), Thailand		
Brunetti (2011), Italy		
Castronovo (2002), Australia		
Corbo (1989), Italy	1.72	1.19 - 2.49
Corbo (2001), Italy		
Ersu (2004), Turkey	3.53	1.34 - 9.30
Gill (2012), New Zealand	1.60	0.90 - 2.90
Gozal (2008), USA		
Kaditis (2004), Greece	1.89	1.35 - 2.64
Kannan (2017), USA	2.00	1.48 - 2.70
Kheirandish-Gozal (2014), Iran	2.25	1.44 - 3.66
Kuehni (2008), UK	1.67	1.43 - 1.95
Li AM (2010), Hong Kong		
Li S (2010), China		
Nosetti (2011), Italy	2.31	2.04 - 2.61
Owen (1996), UK	1.83	1.20 - 2.79
Sahin (2009), Turkey		
Sogut (2005), Turkey		
Sogut (2009), Turkey	1.56	0.85 - 2.87
Urschitz (2004), Germany		
Włodarska (2020), Poland	4.41	1.34 - 14.44
Zhang (2004), Australia	1.62	1.21 - 2.16
Zhu (2013), Hong Kong	1.34	0.95 - 1.88
**Subtotal**	**1.44**	**1.37** - **1.51**

**Fig. 5:**
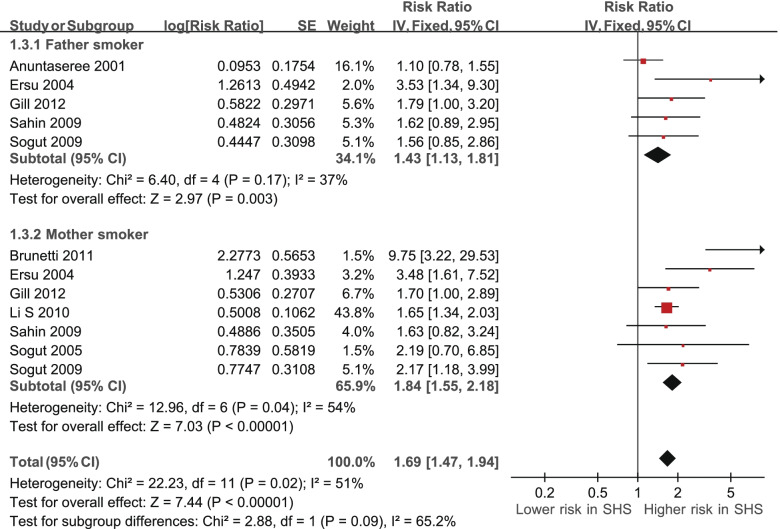
Subgroup analysis of risk ratio of obstructive sleep apnea (OSA) based on smoker fathers and mothers. CI, confidence interval

On the other hand, we analyzed the association between SHS and possible OSA among smoker father and mother subgroups using fixed-effects model meta-analysis of pooled prevalence RRs (Table 4, Fig. 5). For participants with exposure to fathers’ smoking (five RRs were derived from five studies), the pooled prevalence RR of 1.43 (95% CI 1.13 to 1.81, *p* = 0.003) was significant [16, 21, 23, 33, 34]. There was also substantial, not-significant heterogeneity (I^2^ = 37%, χ^2^ = 6.40, *p* = 0.17). For participants with exposure to mothers’ smoking (seven RRs were derived from seven studies), the pooled prevalence RR of 1.84 (95% CI 1.55 to 2.18, *p* < 0.00001) was significant [14, 17, 21, 23, 30, 33, 34]. There was significant heterogeneity (I^2^ = 54%, χ^2^ = 12.96, *p* = 0.04).

**Table 4:** Subgroup analysis of risk ratio based on participants expose to father smoking or mother smoking

Subgroup	Pooled risk ratio	95% confidence interval
**Study participants**		
**Father smoker**		
Anuntaseree (2001), Thailand	1.10	0.78 - 1.57
Ersu (2004), Turkey	3.53	1.34 - 9.30
Gill (2012), New Zealand	1.70	1.00 - 2.80
Sahin (2009), Turkey	1.62	0.89 - 2.93
Sogut (2009), Turkey	1.56	0.85 - 2.87
**Subtotal**	**1.43**	**1.13** - **1.81**
**Mother smoker**		
Brunetti (2011), Italy	9.75	3.22 - 29.56
Ersu (2004), Turkey	3.48	1.61 - 7.51
Gill (2012), New Zealand	1.70	1.00 - 3.00
Li S (2010), China	1.65	1.34 - 2.02
Sahin (2009), Turkey	1.63	0.82 - 3.24
Sogut (2005), Turkey	2.19	0.70 - 6.84
Sogut (2009), Turkey	2.17	1.18 - 4.00
**Subtotal**	**1.84**	**1.55** - **2.18**

## Discussion

This meta-analysis of 26 studies investigated the relationship between SHS and possible OSA. Our analysis, showing an increased risk of possible OSA in the group with exposure to SHS compared to no exposure, confirms exposure to SHS as a risk factor for OSA.

In this meta-analysis, the included articles mostly used cross-sectional study design (except three articles). One [25] of the three articles used a retrospective study design while the other two [27, 31] used prospective cohort and case–control, respectively. The prospective cohort study, performed in the USA in 2017, included participants completing assessment at aged 1–4 and 7 years. Data collection was by complete clinical evaluations and questionnaires. The SHS and non-SHS exposure groups were compared for habitual snoring and the SHS exposure group tends to develop habitual snoring with a RR of 2.00, which imply the significant relationship between possible OSA and SHS exposure, can leads to general understand of the convergence between early SHS exposure and OSA risk.

The comparison of adults and pregnant women were reported in two different studies [22, 32]. According to Ohida et al. [32], significantly higher risk of possible OSA assessed by seven items questionnaire in SHS exposure are found, with an RR of 1.26. On the other hand, in the Franklin et al.’s study [22], habitual snoring affected more people in the passive smoking exposure group than non-exposure group, using Basic Nordic Sleep questionnaire.

Besides, to compare between Asian and USA children, Li et al. [30] administered questionnaires to parents to collect information on Asian children aged from 5–12 years old from the community. Information on snoring frequency and possible correlates according to whether they were exposed or not to household passive smoking were obtained. The household passive smoking exposure group had a higher frequency of habitual snoring than non-exposure group, with an RR of 1.16. On the contrary, Gozal et al. [24] included data on children (boys and girls as well as African American children) aged 5–7 years old collected via parental questionnaire survey regarding whether they were exposed to household smoking or not in the USA. A 1.34 RR of habitual snoring as well as medium to loud snoring was found among exposed children.

On the other hand, 10 studies demonstrated a non-statistically significant association between SHS exposure and the risk of possible OSA [14, 16–18, 23, 29, 33–35, 37]. Limited number of participants might be the reason for the negative results, with only 12.1% of recruited participants taking part. In the largest study conducted in Hong Kong among 9,172 Chinese children aged 5–14 years, the incidence of habitual snoring was demonstrated to be non-significantly different between those with or without household smoking exposure [29]. This result might be due to underreporting of household smoking by parents in the study [29].

OSA, a common sleep-related breathing disorder, is characterized by recurrent episodes of complete or partial reduction of airflow due to obstruction of the upper airway during sleep. It may lead to damage to multiple organs and affects the quality of life. The possible risk factors of OSA include genetic, anatomic, and environmental factors, such as family history of snoring, craniofacial structure, adenotonsillar hypertrophy, chronic allergic rhinitis, neuromuscular control of the upper airway, obesity, and smoking [40–42]. In our study, passive smoking has been reported to be a possible predisposing factor, which facilitates increases in upper airway resistance and pharyngeal collapsibility and leads to intermittent upper airway obstruction during sleep. However, some factors showed no significant association with OSA, including shift work [43] and exposure to solvents [44].

However, the detailed biological mechanisms of this association remain unclear. One potential hypothesis is that chemical-induced pharyngeal inflammation and edema lead to obstruction [45]. Another hypothesis is that SHS exposure may influence neurotransmitters of ventilatory control [46]. The other possible pathological change is that the long-term exposure to SHS reduces the sensitivity of tissues to hypoxia and impair the ability to recover from conditions caused by hypoxia [47, 48]. Besides, nicotine also plays a role in the progression of possible OSA [49]. The possible mechanism is through acetylcholine stimulation and activation of dopaminergic pathways in the central neurologic system [50].

In our meta-analysis, most of the enrolled studies use questionnaire surveys to detect possible OSA [16, 18–24, 26–30, 32–39], and a few use PSG [13, 14, 17, 25, 31]. PSG is the gold standard for diagnosing OSA as recommended by the American Academy of Pediatrics and American Association of Sleep Medicine [51]. However, PSG is expensive and not always available in most areas. Hence, many screening tools were developed for OSA, including the Berlin questionnaire [52], pediatric sleep questionnaire (PSQ) [53], and Children's Sleep Habits Questionnaire (CSHQ) [54], and all the three tools are powerful to detect possible OSA.

As we know, clinical manifestations, risk factors, diagnostic criteria, and polysomnographic findings of OSA in children are likely different from those of adults. The most common underlying condition in adults is often obesity, while those in children include enlargement of adenoids and tonsils. According to the third edition of the International Classification of Sleep Disorders (ICSD-3), OSA is divided into adult and pediatric OSA [55]. In our meta-analysis, two groups (for adults and children) were examined as part of our objectives. Although the mechanism of the cause of OSA was not totally similar in the two groups, the outcome was identical. Three out of 26 articles reported on the adult group, with an RR of 1.35. The remaining articles reported on the children’s group, with an RR of 1.44. Interestingly, the children group presented with more statistically significantly different and higher RRs than those of the adult group.

The subgroup analysis revealed that SHS, following smoker father or mother subgroups, has congruent effects on children's OSA. The present study showed that mothers smoking was a significantly higher risk of children’s OSA compared to fathers smoking. The possible reason is because mothers are the children’s main caregivers and spend more time caring for the children. Bianchi et al. reported that mothers, compared to fathers, spend two-fold time (120 vs. 60 min per day) caring for the children [56]. In Sogut et al., a significant difference was found between fathers smoking, compared with mothers smoking, and children’s snoring rate [34]. This was attributed to the fact that children spend more time with their mothers in Turkey [34]. However, few studies have examined the impact of mothers or fathers smoking on possible OSA occurrence, and more studies are needed for more precise results in the future.

### Limitations

Our study has several limitations that inevitably exist in the meta-analysis. First, most of the studies enrolled were cross-sectional studies, only one was a prospective cohort study. The primary finding of the present study have shown a significant result on the relationship between SHS and possible OSA, however due to the heterogeneity in the included participants groups, experiment designs and study type. The finding of the present study can be equivocal, the paucity of prospective cohort studies and the SHS exposure quantity will need to be further addressed to clearly establish the relationship between SHS and OSA. Therefore, additional prospective cohort studies are needed to confirm the causal relationship between SHS and possible OSA. Second, the definition of SHS exposure for each enrolled study differed, which made it difficult to quantify the SHS exposure. More definitive definition or quantification of SHS will be required in the future. Third, the outcome measurements among studies were inconsistent. Only three studies measured using PSG, most studies measured using questionnaires; this might induce recall bias. More objective measurements are required to improve the reliability of these studies. Fourth, the definitions of possible OSA (habitual snoring) were different among the studies. Some studies reported for every night, more than four times a week, or more than three times a week. Furthermore, some studies had no clear limit on the number of times, they only reported the degree (i.e., sometimes, often, and always). A more accurate and effective questionnaire for OSA assessment need to be considered in the future study. Fifth, the other risk factors, such as hypertension, diabetes mellitus, gender, advancing age, and body weight, did not be adjusted in the meta-analysis. Adjusting the same and consisted confounding variables in meta-analysis study is impossible because the diversity of confounding variables in these included original studies. Sixth, we did not analyze certain internal biomarkers (such as cotinine or similar) because all included studies were lacking these biomarkers expect only Zhu’s study [37], which applied urine cotinine as an objective biomarkers for quantifying SHS exposure. Determining the association of internal biomarkers (such as cotinine or similar) with OSA may be a topic for future research.

## Conclusion

Our meta-analysis revealed a significant and positive association between SHS exposure and possible OSA in both children and adults. Moreover, both mothers smoking and father smoking are associated with a significantly higher risk of possible OSA in children compared nonsmoking in parents. However, the possible mechanism requires further survey.

## Data Availability

Data will be available upon request after the paper is accepted.

## Abbreviations

CI: Confidence interval
OSA: Obstructive sleep apnea
PSG: Polysomnography
RR: Risk ratio
SE: Standard error
SHS: Secondhand smoke

## References

[1] Yaggi HK, Strohl KP (2010). Adult obstructive sleep apnea/hypopnea syndrome: definitions, risk factors, and pathogenesis. Clin Chest Med.

[2] Bonsignore MR, Saaresranta T, Riha RL (2019). Sex differences in obstructive sleep apnoea. Eur Respir Rev..

[3] Wu KL, Kuo CY, Tsai YC, Hung JY, Sheu CC, Yang CJ (2019). CHADS_2_, CHA_2_DS_2_ASc, and New ABCD scores predict the risk of peripheral arterial disease in patients with sleep apnea. J Clin Med..

[4] Hsu WY, Chiu NY, Chang CC, Chang TG, Lane HY (2019). The association between cigarette smoking and obstructive sleep apnea. Tob Induc Dis.

[5] Wang X, Ouyang Y, Wang Z, Zhao G, Liu L, Bi Y (2013). Obstructive sleep apnea and risk of cardiovascular disease and all-cause mortality: a meta-analysis of prospective cohort studies. Int J Cardiol.

[6] Ge X, Han F, Huang Y, Zhang Y, Yang T, Bai C (2013). Is obstructive sleep apnea associated with cardiovascular and all-cause mortality?. PLoS ONE.

[7] World Health Organization (2017). WHO report on the global tobacco epidemic, 2017: monitoring tobacco use and prevention policies.

[8] Carter BD, Abnet CC, Feskanich D, Freedman ND, Hartge P, Lewis CE (2015). Smoking and mortality–beyond established causes. N Engl J Med.

[9] DiGiacomo SI, Jazayeri MA, Barua RS, Ambrose JA (2018). Environmental tobacco smoke and cardiovascular disease. Int J Environ Res Public Health..

[10] Roberts C, Wagler G, Carr MM (2017). Environmental tobacco smoke: public perception of risks of exposing children to second- and third-hand tobacco smoke. J Pediatr Health Care.

[11] Szukalska M, Merritt TA, Lorenc W, Sroczyńska K, Miechowicz I, Komorowicz I (2021). Toxic metals in human milk in relation to tobacco smoke exposure. Environ Res.

[12] Krishnan V, Dixon-Williams S, Thornton JD (2014). Where there is smoke there is sleep apnea: exploring the relationship between smoking and sleep apnea. Chest.

[13] Włodarska A, Doboszyńska A (2020). Tobacco smoke exposure as a risk factor for obstructive sleep apnea in children. Pediatr Int.

[14] Sogut A, Altin R, Uzun L, Ugur MB, Tomac N, Acun C (2005). Prevalence of obstructive sleep apnea syndrome and associated symptoms in 3–11-year-old Turkish children. Pediatr Pulmonol.

[15] Hunter JE, Schmidt FL (2000). Fixed effects vs. random effects meta-analysis models: implications for cumulative research knowledge. Int J Sel Assess.

[16] Anuntaseree W, Rookkapan K, Kuasirikul S, Thongsuksai P (2001). Snoring and obstructive sleep apnea in Thai school-age children: prevalence and predisposing factors. Pediatr Pulmonol.

[17] Brunetti L, Rana S, Lospalluti ML, Pietrafesa A, Francavilla R, Fanelli M (2001). Prevalence of obstructive sleep apnea syndrome in a cohort of 1,207 children of southern Italy. Chest.

[18] Castronovo V, Zucconi M, Nosetti L, Marazzini C, Hensley M, Veglia F (2003). Prevalence of habitual snoring and sleep-disordered breathing in preschool-aged children in an Italian community. J Pediatr.

[19] Corbo GM, Forastiere F, Agabiti N, Pistelli R, Dell'Orco V, Perucci CA (2001). Snoring in 9- to 15-year-old children: risk factors and clinical relevance. Pediatrics.

[20] Corbo GM, Fuciarelli F, Foresi A, De Benedetto F (1989). Snoring in children: association with respiratory symptoms and passive smoking. BMJ.

[21] Ersu R, Arman AR, Save D, Karadag B, Karakoc F, Berkem M (2004). Prevalence of snoring and symptoms of sleep-disordered breathing in primary school children in istanbul. Chest.

[22] Franklin KA, Gíslason T, Omenaas E, Jõgi R, Jensen EJ, Lindberg E (2004). The influence of active and passive smoking on habitual snoring. Am J Respir Crit Care Med.

[23] Gill AI, Schaughency E, Galland BC (2012). Prevalence and factors associated with snoring in 3-year olds: early links with behavioral adjustment. Sleep Med.

[24] Gozal D, Kheirandish-Gozal L, Capdevila OS, Dayyat E, Kheirandish E (2008). Prevalence of recurrent otitis media in habitually snoring school-aged children. Sleep Med.

[25] Huang Q, Ali S, Zhang J (2019). Association between exposure to family environmental tobacco smoke and obstructive sleep apnea hypopnea syndrome. Appl Ecol Environ Res.

[26] Kaditis AG, Finder J, Alexopoulos EI, Starantzis K, Tanou K, Gampeta S (2004). Sleep-disordered breathing in 3,680 Greek children. Pediatr Pulmonol.

[27] Kannan JA, Brokamp C, Bernstein DI, LeMasters GK, Hershey GKK, Villareal MS (2017). Parental snoring and environmental pollutants, but not aeroallergen sensitization, are associated with childhood snoring in a birth cohort. Pediatr Allergy Immunol Pulmonol.

[28] Kheirandish-Gozal L, Ghalebandi M, Salehi M, Salarifar MH, Gozal D (2014). Neighbourhood air quality and snoring in school-aged children. Eur Respir J.

[29] Li AM, Au CT, So HK, Lau J, Ng PC, Wing YK (2010). Prevalence and risk factors of habitual snoring in primary school children. Chest.

[30] Li S, Jin X, Yan C, Wu S, Jiang F, Shen X (2010). Habitual snoring in school-aged children: environmental and biological predictors. Respir Res.

[31] Caprioglio A, Levrini L, Nosetti L, Berini J, Macchi A, Tagliabue A (2011). Prevalence of malocclusion in preschool and primary school children with habitual snoring and sleep-disordered breathing. Eur J Paediatr Dent.

[32] Ohida T, Kaneita Y, Osaki Y, Harano S, Tanihata T, Takemura S (2007). Is passive smoking associated with sleep disturbance among pregnant women?. Sleep.

[33] Sahin U, Ozturk O, Ozturk M, Songur N, Bircan A, Akkaya A (2009). Habitual snoring in primary school children: prevalence and association with sleep-related disorders and school performance. Med Princ Pract.

[34] Sogut A, Yilmaz O, Dinc G, Yuksel H (2009). Prevalence of habitual snoring and symptoms of sleep-disordered breathing in adolescents. Int J Pediatr Otorhinolaryngol.

[35] Urschitz MS, Guenther A, Eitner S, Urschitz-Duprat PM, Schlaud M, Ipsiroglu OS (2004). Risk factors and natural history of habitual snoring. Chest.

[36] Zhang G, Spickett J, Rumchev K, Lee AH, Stick S (2004). Snoring in primary school children and domestic environment: a Perth school based study. Respir Res.

[37] Zhu Y, Au CT, Leung TF, Wing YK, Lam CW, Li AM (2013). Effects of passive smoking on snoring in preschool children. J Pediatr.

[38] Owen G, Canter R, Robinson A (1996). Snoring, apnoea and ENT symptoms in the paediatric community. Clin Otolaryngol.

[39] Kuehni CE, Strippoli MP, Chauliac ES, Silverman M (2008). Snoring in preschool children: prevalence, severity and risk factors. Eur Respir J.

[40] Włodarska A, Doboszynska A (2016). Obstructive sleep apnoea syndrome in children. Pediatria i Medycyna Rodzinna.

[41] Schwengel DA, Dalesio NM, Stierer TL (2014). Pediatric obstructive sleep apnea. Anesthesiol Clin.

[42] Kent DT, Soose RJ (2014). Environmental factors that can affect sleep and breathing: allergies. Clin Chest Med.

[43] Yang CC, Lee KW, Watanabe K, Kawakami N (2021). The association between shift work and possible obstructive sleep apnea: a systematic review and meta-analysis. Int Arch Occup Environ Health.

[44] Schwartz DA, Vinnikov D, Blanc PD (2017). Occupation and obstructive sleep apnea: a meta-analysis. J Occup Environ Med.

[45] Weinstock TG, Rosen CL, Marcus CL, Garetz S, Mitchell RB, Amin R (2014). Predictors of obstructive sleep apnea severity in adenotonsillectomy candidates. Sleep.

[46] Schuetze P, Eiden RD (2007). The association between prenatal exposure to cigarettes and infant and maternal negative affect. Infant Behav Dev.

[47] Coggins CR (2010). A further review of inhalation studies with cigarette smoke and lung cancer in experimental animals, including transgenic mice. Inhal Toxicol.

[48] Owili PO, Muga MA, Kuo HW (2018). Gender difference in the association between environmental tobacco smoke and birth weight in Africa. Int J Environ Res Public Health..

[49] Law KL, Stroud LR, LaGasse LL, Niaura R, Liu J, Lester BM (2003). Smoking during pregnancy and newborn neurobehavior. Pediatrics.

[50] Stroud LR, Paster RL, Papandonatos GD, Niaura R, Salisbury AL, Battle C (2009). Maternal smoking during pregnancy and newborn neurobehavior: effects at 10 to 27 days. J Pediatr.

[51] Aurora RN, Zak RS, Karippot A, Lamm CI, Morgenthaler TI, Auerbach SH (2011). Practice parameters for the respiratory indications for polysomnography in children. Sleep.

[52] Netzer NC, Stoohs RA, Netzer CM, Clark K, Strohl KP (1999). Using the Berlin questionnaire to identify patients at risk for the sleep apnea syndrome. Ann Intern Med.

[53] Chervin RD, Hedger K, Dillon JE, Pituch KJ (2000). Pediatric sleep questionnaire (PSQ): validity and reliability of scales for sleep-disordered breathing, snoring, sleepiness, and behavioral problems. Sleep Med.

[54] Owens JA, Spirito A, McGuinn M (2000). The Children's Sleep Habits Questionnaire (CSHQ): psychometric properties of a survey instrument for school-aged children. Sleep.

[55] Sateia MJ (2014). International classification of sleep disorders-third edition: highlights and modifications. Chest.

[56] Bianchi SM (2011). Family Change and Time Allocation in American Families. Ann Am Acad Pol Soc Sci.

